# Antioxidant activity of the stem bark of *Shorea roxburghii* and its silver reducing power

**DOI:** 10.1186/2193-1801-2-28

**Published:** 2013-01-29

**Authors:** Ramasamy Subramanian, Palanivel Subbramaniyan, Vairamuthu Raj

**Affiliations:** 1 Advanced Materials Research Laboratory, Department of Chemistry, Periyar University, Salem, 636011 Tamil Nadu India; 2 Department of Chemistry, Sona College of Technology, Salem, 636005 Tamil Nadu India

**Keywords:** DPPH, Hydroxyl radical, Hydrogen peroxide, Ferric reducing power, Shorea roxburghii, Silver nanoparticles

## Abstract

A detailed study has been performed on the antioxidant activity of the acetone and methanol extracts of the stem bark of the plant, *Shorea roxburghii*. The total phenolic content and antioxidant activity of the extracts were determined by DPPH, radical scavenging, ferric ion reducing power, hydroxyl radical, ABTS^.^ radical scavenging and hydrogen peroxide scavenging activities. Reducing efficiency of the *S. roxburghii* towards silver nanoparticles has been evaluated using surface plasmon resonance and transmission electron microscope. Spherical shapes of particles with 4–50 nm have been reported. Formation of silver nanoparticles ascertains the role of the water soluble phenolic compounds present in *S. roxburghii*. Both acetone and methanol extracts of *S. roxburghii* stem bark was found to be a potent antioxidant. This work provides a scientific support for the high antioxidant activity of this plant and thus it may find potential applications in the treatment of the diseases caused by free radical. The extract of this plant could be used as a green reducing agent for the synthesis of Ag nanoparticles.

## Introduction

There has been intense interest recently among the public and the media in the possibility that increased intake of dietary antioxidants may protect against chronic diseases, which include cancers, cardiovascular, and cerebrovascular diseases. Antioxidants are substances that, when present at low concentrations, compared with those of an oxidizable substrate, significantly prevent or delay a pro-oxidant–initiated oxidation of the substrate (Prior and Cao, 1999). A pro-oxidant is a toxic substance that can cause oxidative damage to lipids, proteins, and nucleic acids, resulting in various pathological events or diseases. Examples of pro-oxidants include reactive oxygen and nitrogen species (ROS and RNS), which are products of normal aerobic metabolic processes. ROS include superoxide (O_2_−·), hydroxyl (OH·), and peroxyl (ROO·) radicals, and hydrogen peroxide (H_2_O_2_). RNS include nitric oxide (NO·) and nitrogen dioxide (NO_2_·) (Gülçin, 2012; Gülçin *et al*. 2011). There is a considerable biological evidence that ROS and RNS can be damaging to cells and, thereby, they might contribute to cellular dysfunction and diseases. The existence and development of cells in an oxygen-containing environment would not be possible without the presence of a complicated antioxidant defense system that includes enzymatic and nonenzymatic components. The nonenzymatic antioxidants, most of which have low molecular weights and are able to directly and efficiently quench ROS and RNS, constitute an important aspect of the body’s antioxidant system components (Cao and Prior, 2002). The interaction among these antioxidants and the difficulty in measuring all of them individually prompted the development of assays for measuring total antioxidant capacity. The measurement of total antioxidant capacity of all these nonenzymatic antioxidants is necessary and important in evaluating in vivo antioxidant status in many clinical and nutritional studies.

*Shorea roxburghii* is a semievergreen endangered tree which grows up to 100 m, fall on the slope of the hill area of peninsular India, which is included in the list of medicinal plants of conservation areas of Eastern and Western Ghats of South India (Rani and Pullaiah, 2002) especially in Kolli Hills of central Tamil Nadu (Matthew, 1999) and some population are distributed in Alagar Hills of Madurai (Karuppusamy *et al.*^2009^). The genus *shorea* is a rich source for oligomeric stilbenes (Sotheeswaran and Pasupathy, 1993). The bark of *S. roxburghii* has been used as an astringent or a preservative for traditional beverages in Thailand. In Indian folk medicine, it has been used for treatments of dysentery, diarrhoea, and cholera (Chitravadivu *et al.*^2009^). Previous phytochemical study of the *Shorea* species revealed the presence of various stilbenoids (Tukiran *et al.*^2005^). Some of these stilbenoids which show interesting biological activities such as cytotoxic (Seo *et al.*^1999^& Aminah *et al.*^2002^), antioxidant (Tanaka *et al.*^2000^; Saisin *et al.*^2009^), antiplatelet aggregation (Aburjai, 2000) and cyclooxygenase inhibitory activities (Li *et al.*^2001^). The aim of the present study is to explore the antioxidant potential of acetone and methanol extracts of stem bark of *Shorea roxburghii*. DPPH, ABTS, hydroxyl radical and hydrogen peroxide scavenging activities and ferric reducing power assays have been to evaluate the antioxidant activity of these extracts. The reducing ability of water extract towards Ag nanoparticles has been also evaluated.

## Materials and methods

### Chemicals

1, 1-diphenyl-2-picrylhydrazyl radical and ABTS radicals were purchased from Sigma-Aldrich, Bangalore, India. Gallic acid, trichloroacetic acid, potassium ferricyanide, ferric chloride, aluminium chloride, Folin-Ciocalteau reagent (phenol reagent), methanol, sodium carbonate, sodium hydroxide, sodium nitrite, ammonium acetate, acetone, glacial acetic acid, ascorbic acid (vitamin C), ferrous ammonium sulphate, EDTA, DMSO, potassium persuphate and silver nitrate (AgNO_3_) were procured from Merck, India. All chemicals used were of analytical grade and used as such without further purification. All the solutions were prepared with Millipore water.

### Plant collection and preparation of crude extract

Stem bark of *Shorea roxburghii* was collected from Alagar Hills, Madurai, Tamil Nadu, India during March 2010. The plant materials were dried under shade, pulverized and used for the preparation of crude extract. 50 g of powdered stem bark materials of *S. roxburghii* in the thimble were introduced into double bypass soxhlet apparatus (DBSA) which was connected with two distillation flasks through inverted Y shaped joints and extracted with 500 mL of acetone and methanol (Subramanian *et al.*^2011^). The extracts were evaporated to dryness under reduced pressure in a rotary evaporator. The obtained acetone (8 g) and methanol 6 (g) crude extracts were used for the measurements of total phenolic content and antioxidant activities. 20 g of powdered stem bark of *S. roxburghii* was extracted with boiling water for 30 min and evaporated to dryness in a water bath. The obtained crude extract was used for the synthesis of silver nanoparticles.

### Total phenolic content

Total phenolic content of acetone and methanol extracts of stem bark of *S.roxburghii* was determined by the method of Singletone and Rossi, 1965. 10 mg of individual plant extract was dissolved in methanol to get the appropriate concentration (1 mg/mL). 1.0 mL of each extract in a test tube was mixed with 5.0 mL of distilled water. 1.0 mL of Folin-Ciocalteau reagent was added and mixed thoroughly. 3 min later, 3.0 mL of saturated sodium carbonate solution was added and the mixture was allowed to stand for 90 min in the dark. The absorbance of the color developed was read at 725 nm using UV–Vis spectrophotometer. The concentration of total phenolic content in the extracts was determined as μg of gallic acid equivalent (GAE) by calibration curve (r^2^=0.989). Three replicates were performed for each sample concentration to check the reproducibility of the experimental result and to get a more accurate result. The results were represented as mean ± standard deviation.

### Antioxidant activity assay

#### DPPH radical scavenging activity

Various concentrations (20, 40, 60, 80 & 100 μg/mL) of extracts were mixed with 3.0 mL of methanolic solution containing DPPH radical (6×10^-5^ mol/L). The mixture was shaken vigorously and left to stand for 60 min in the dark. The reduction of the DPPH radical was determined by recording the absorbance at 517 nm using UV–Vis spectrophotometer. DPPH radical-scavenging activity was calculated by the following equation:

where A_DPPH_ is the absorbance without samples and A_s_ the absorbance in the presence of the samples. A lower absorbance of the reaction mixture indicated a higher DPPH radical-scavenging activity (Gülçin, 2003; Gülçin, 2011).

#### Ferric reducing power

Exactly 1 mL of the extract was mixed with 2.5 mL of phosphate buffer (0.2 M, pH 6.6) and 2.5 mL of potassium ferricyanide (1%). The mixture was incubated at 50°C for 30 min. Afterwards, 2.5 mL of trichloroacetic acid (10%) was added to the mixture, which was then centrifuged at 3000 rpm for 10 min. Finally, 2.5 mL of the upper layer was pipetted out and mixed with 2.5 mL of distilled water and 0.5 mL of ferric chloride (0.1%) was added. The absorbance was measured at 700 nm using a Perkin Elmer Lambda 35 UV-Visible Spectrophotometer. The intensity of reducing power is directly proportional to the absorbance of the reaction mixture (Barrors *et al.*^2007^; Yildirim *et al.*^2001^).

#### Hydrogen peroxide radical scavenging activity

The ability of the extracts to scavenge hydrogen peroxide was determined according to the method of Cetinkaya *et al.*^2012^. Hydrogen peroxide solution (1 mM/L) was prepared with 50 mM phosphate buffer (pH 7.4). Different concentrations (20–100 μg) of the extracts (1 mL) were allowed to react with 0.6 mL of hydrogen peroxide solution. Absorbance was determined at 230 nm after 10 min against a blank solution containing phosphate buffer without hydrogen peroxide. Hydrogen peroxide scavenging activity was calculated according to the following equation:

where A_c_ is the absorbance without samples and A_s_ is the absorbance in the presence of the samples.

#### ABTS radical scavenging activity

ABTS radical scavenging activity was estimated by the method of Bursal and Gülçin 2011and Gülçin *et al*. 2010. The stock solutions included 7 mM ABTS solution and 2.4 mM potassium persuphate solution. The working solution was then prepared by mixing the two stock solutions in equal quantities and allowing them to react for overnight at room temperature in the dark. The solution was then diluted by mixing 1 mL of ABTS solution with 60 mL ethanol to obtain an absorbance of 0.706 ± 0.001 units at 734 nm. Fresh ABTS solution was prepared for each assay. Different concentrations (20–100 μg) of the extracts (1 mL) were allowed to react with 1 mL of the ABTS solution and the absorbance was measured at 734 nm after 7 min using a Perkin Elmer Lambda 35 UV-Visible Spectrophotometer. ABTS radical scavenging activity was calculated according to the following equation:

where A_c_ is the absorbance without samples and A_s_ the absorbance in the presence of the samples.

#### Hydroxyl radical scavenging activity

Hydroxyl radical scavenging activity of the extracts was carried out by the method of Halliwell and Gutteridge 1981. Exactly, 0.2 mL of the extract was added with 1.0 mL of EDTA solution (0.13 g of ferrous ammonium sulphate and 0.26 g of EDTA were dissolved in 100 mL of water) and mixed with 1.0 mL of DMSO (0.85%) in 0.1 M phosphate buffer (pH 7.4) to initiate the reaction followed by the addition of 0.5 mL of 0.22% ascorbic acid. The reaction mixture was kept in a water bath at 90°C for 15 min and the reaction was terminated by adding 1.0 mL of ice-cold 17.5% trichloroacetic acid. Further 3.0 mL of Nash reagent (75 g of ammonium acetate, 3.0 mL of glacial acetic acid and 2.0 mL of acetyl acetone in 1.0 L of water) was added to all the test tubes and incubated for 15 min for color development. The absorbance was observed at 412 nm. The reaction mixture without ascorbic acid served as control. The ability to scavenge hydroxyl radical was calculated by the following equation:

where A_c_ is the absorbance without samples and A_e_ the absorbance in the presence of the samples.

EC_50_ value (μg extract/mL) is the effective concentration at which the reducing power, hydrogen peroxide, DPPH, ABTS radical and hydroxyl radical scavenging activities were scavenged by 50% and were obtained by interpolation from linear regression analysis. Vitamin C was used as a standard.

#### Synthesis and characterization of silver nanoparticles

Reducing ability of the stem bark extract of *S.roxburghii* in the formation of silver nanoparticles (AgNPs) from silver nitrate was tested using *S. roxburghii* as reducing and capping agent. 20 g of the powdered stem bark of *S. roxburghii* was boiled in water and used as reducing agent. Various concentrations of extract (1.0, 2.5, 3.5 and 5.0 mL) were added drop wise to 50 mL of silver nitrate solution (1.0 mM) with constant stirring. The kinetics of the reaction was monitored by measuring the surface plasmon resonance (SPR) of the reaction mixture at different time intervals by UV-Visible spectrophotometer. The shape and size of the particles were measured with high resolution transmission electron microscopy (TEM) using JEOL JEM-100CX II equipped with selected area electron diffraction pattern (SAED).

### Statistical analysis

Triplicate analysis were performed by excel sheet. The results were presented as the mean ± S.D. Statistical analysis was performed using student’s *t*-test and a P < 0.05 was regarded to be significant.

## Results and discussion

### Total phenol content

**Figure 1:**
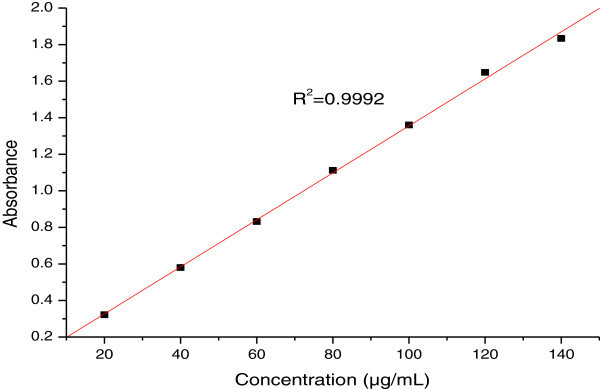
**Calibration curve for gallic acid (20–140 μg/mL).**

The amount of total phenols present in acetone and methanol extract of stem bark of *S.roxburghii* was determined from the regression equation (y = ax + b) of calibration curve of gallic acid standard solution and expressed in gallic acid equivalents (Figure 1). Total phenolic content of acetone (65.74 ± 8.70 μg/mL) and methanol (67.67 ± 4.90 μg/mL) extracts were found to be similar. From the results it can be seen that the extraction ability of acetone and methanol are very similar to one another. Phenolics are secondary plant metabolites that are present in every plant and plant product. Many of the phenolics have been shown to contain high levels of antioxidant activities. Phenolic compounds present in the plants acting as antioxidant or free radicals scavengers (Kahkonen *et al.*^1999^) due to their hydroxyl groups which contribute directly to the antioxidative action (Diplock, 1997). Phenolic compounds are effective hydrogen donors, making them good antioxidants (Rice-Evan and Miller 1997).

### DPPH radical scavenging activity

The radical-scavenging activity of the acetone and methanol extracts of *S. roxburghii* was estimated by comparing the percentage inhibition of formation of DPPH radicals with that of vitamin C. Both acetone and methanol extracts showed moderate antioxidant activity when compared with Vitamin C. The DPPH radical scavenging activity of acetone and methanol extracts increased with increasing the concentration (Table 1). Our results were in agreement with Ragini *et al.*^2011^who reported radical scavenging activity of 23.40, 34.50, 48.67, 65.40 and 79.50% in ethanolic extract of *Shorea tumbuggaia* at a *concentration* of 20, 40, 60, 80 and 100 μg/mL, respectively. Natural antioxidants those are present in medicinal plants which are responsible for inhibiting the harmful consequences of oxidative stress. Many plants extract exhibit efficient antioxidant properties due to their phytoconstituents, including phenolics (Larson, 1988). This method has been extensively used for screening antioxidants, such as polyphenols. The antioxidant effectiveness in natural sources has been reported to be mostly due to phenolic compounds. Phenolic compounds may contribute directly to antioxidative effect of the extracts. The free radical scavenging activity of acetone and methanolic extracts were confirmed in the present investigation.

**Table 1:** **Radical scavenging activities of the stem bark extract of*S. roxburghii***

	DPPH radical scavenging activity (%)					
Concentrations	SAE	IC_50_	SME	IC_50_	Vitamin C	IC_50_
μg/mL		(μg/mL)		(μg/mL)		(μg/mL)
20	26.96 ± 0.09		19.75 ± 0.15		12.67 ± 0.06	
40	29.56 ± 0.12		26.26 ± 0.07		44.16 ± 0.21	
60	34.37 ± 0.15	na*	31.72 ± 0.09	na*	74.42 ± 0.12	45.74
80	39.33 ± 0.15		38.54 ± 0.12		89.51 ± 0.09	
100	43.94 ± 0.12		42.78 ± 0.15		97.13 ± 0.06	
	Hydroxyl radical scavenging activity (%)					
20	29.89 ± 0.59		28.80 ± 0.40		30.46 ± 0.78	
40	44.97 ± 0.13		47.80 ± 0.30		44.44 ± 0.39	
60	52.59 ± 0.27	52.56	58.08 ± 0.08	50.93	63.66 ± 0.26	45.91
80	67.41 ± 0.46		65.45 ± 0.20		73.73 ± 0.52	
100	75.16 ± 0.35		74.25 ± 0.92		86.27 ± 0.13	
	ABTS radical scavenging activity (%)					
20	20.77 ± 0.92		19.60 ± 1.40		28.28 ± 1.20	
40	45.07 ± 0.61		19.60 ± 1.40		46.40 ± 0.40	
60	56.13 ± 1.01	55.24	58.13 ± 0.61	55.96	69.73 ± 0.92	43.05
80	65.20 ± 0.80		64.40 ± 0.40		83.47 ± 0.46	
100	79.20 ± 1.06		86.27 ± 1.22		97.96 ± 0.04	
	Hydrogen peroxide scavenging activity (%)					
20	24.24 ± 0.38		15.65 ± 0.60		41.88 ± 0.68	
40	32.34 ± 0.30		35.22 ± 0.62		58.17 ± 0.56	
60	46.45 ± 0.23	87.18	50.27 ± 0.31	63.67	72.33 ± 0.52	28.53
80	25.77 ± 0.34		63.39 ± 0.52		81.27 ± 0.31	
100	67.06 ± 0.45		73.47 ± 0.39		90.71 ± 0.38	
	Ferric reducing power (OD)*					
20	0.078 ± 0.008		0.139 ± 0.006		0.216 ± 0.002	
40	0.165 ± 0.004		0.235 ± 0.005		0.387 ± 0.005	
60	0.205 ± 0.007	na*	0.347 ± 0.007	na*	0.568 ± 0.007	52.19
80	0.258 ± 0.008		0.431 ± 0.001		0.767 ± 0.013	
100	0.376 ± 0.006		0.479 ± 0.009		0.918 ± 0.007	

OD* Increasing the optical density: na*-Not available.

DPPH is a stable free radical at room temperature and accepts an electron or hydrogen radical to become stable diamagnetic molecules (Soares *et al.*^1997^). This method has been extensively used for screening antioxidants, such as polyphenols. DPPH radical is scavenged by polyphenols through donation of hydrogen, forming the reduced form of DPPH. Then the colour changes from purple to yellow after reduction, which can be quantified by its decrease absorbance at wavelength 517 nm (Amarowicz *et al.*^2004^; Bondet *et al.*^1997^). The decrease in absorbance of DPPH radical caused by antioxidants, because of the reaction between antioxidant molecule and radical progresses, results in the scavenging of the radical by hydrogen donation. It is visually noticeable as a discoloration from purple to yellow. These results revealed that the acetone and methanol extracts of *S roxburghii* is free radical inhibitor or scavenger acting possibly as primary antioxidants.

### Hydroxyl radical scavenging activity

Hydroxyl radical inhibition of *S. roxburghii* was investigated and these results are shown as relative activity against the standard (Vitamin C). Hydroxyl radical scavenging activity of *S. roxburghii* is presented in Table 1. There is no significant difference (p > 0.05) in the hydroxyl radical scavenging activities of the acetone and methanol extracts, showing that these extracts are equally potent in scavenging hydroxyl radicals. The extracts were less effective in comparison with vitamin C. IC_50_ values were 52.56 and 50.93 μg/mL, whereas that of vitamin C was 45.91 μg/mL. Dose-dependent hydroxyl radical scavenging activity reveals that, acetone and methanol extracts of *S. roxburghii* have potent hydroxyl radical scavengers, acting possibly as primary antioxidants. Hydroxyl radical is an extremely reactive free radical formed in biological system and has been implicated as a highly damaging species in free radical pathology, capable of damaging almost every molecule, proteins, DNA, unsaturated fatty acids and lipids in almost every biological membranes found in living cells (Hochestein and Atallah, 1988; Rollet-Labelle *et al.*^1998^; Trease and Evans, 1983).

### ABTS Radical scavenging activity

Both acetone and methanol extracts showed comparable scavenging effects on ABTS^·+^. The extracts were less effective in comparison with vitamin C. The ABTS radical scavenging activity of the acetone extract (20.77, 45.07, 56.13, 65.20 and 79.20%) was comparable with that of methanol extract (19.60, 36.27, 58.13, 64.40 and 86.27%). IC_50_ values for acetone and methanol extracts were 55.24 and 55.96 μg/mL, respectively whereas that of vitamin C was 43.05 μg/mL (Table 1). The results clearly imply that the acetone and methanol extracts of *S. roxburghii* inhibit ABTS radical or scavenge the radical in a dose dependent manner. ABTS^·+^ radical is generated from oxidation of ABTS^·+^ by potassium persulphate, is a good tool for determining the antioxidant activity of hydrogen-donating and chain breaking antioxidants (Leong and Shui, 2002). This assay is applicable for both lipophilic and hydrophilic antioxidants. The radical-scavenging activity of the acetone and methanol extracts of *S. roxburghii* were estimated by comparing the percentage inhibition of formation of ABTS^·+^ radicals with that of vitamin C. These extracts exhibited the highest radical-scavenging activities when reacted with the ABTS radicals.

### Hydrogen peroxide radical scavenging activity

The hydrogen peroxide radical-scavenging activity of the acetone and methanol extracts of *S. roxburghii* was estimated by comparing the percentage inhibition of formation of peroxyl radicals with that of vitamin C. Hydrogen peroxide scavenging activity of acetone and methanol extracts of *S. roxburghii* are presented in Table 1. Both acetone and methanol extracts showed moderate inhibition against peroxyl radical which was less in comparison with vitamin C. These results showed that acetone and methanol extracts of *S. roxburghii* are highly potent in neutralizing hydrogen peroxide radicals. Most of the hydrogen peroxide was scavenged by the extracts. IC_50_ values for acetone and methanol extracts were 87.18 and 63.67 μg/mL, respectively whereas that of Vitamin C was 28.53 μg/mL. H_2_O_2_ itself is not very reactive, but it can sometimes be toxic to cell because it may give rise to hydroxyl radical in the cells. The results showed that *S. roxburghii* extracts have an effective H_2_O_2_ scavenging activity.

### Ferric reducing power

In the reducing power assay, the presence of antioxidants in the extract of *S. roxburghii* would result in the reduction of Fe^3+^/ferricyanide complex to its form. The reducing power of compound may serve as a significant indicator of its potential antioxidant activity (Meir *et al.*^1995^). The ferric reducing power of the acetone and methanol extracts of *S. roxburghii* was determined by comparing with that of vitamin C. The increased absorbance values of the extracts at 700 nm indicate an increase in reductive ability. Absorbance values of acetone and methanol extracts are presented in Table 1. Ferric reducing power increasing the absorbance values Absorbance values of acetone and methanol extracts are presented in Table 1. The reducing power of ascorbic acid was found to be significantly higher than those of acetone and methanol extracts. In this assay, the yellow color of the test solution was changed to various shades of green and blue depending on the reducing power of each compound. At 1000 μg/mL, the reducing powers of the acetone and methanol extract were 0.078–0.378 and 0.139–0.479, respectively whereas that of vitamin C was 0.216–0.918. The reducing power increased with increasing the phenolic content of the extract. It was found that the reducing powers of all the extracts also increased with the increase of their concentrations. This data imply that these extracts have significant ability to react with free radicals to convert them into more stable nonreactive species and to terminate radical chain reaction.

Numerous methods are available to evaluate of antioxidant activity. For in vitro antioxidant screening, DPPH, ABTS, hydroxyl radical scavenging, hydrogen peroxide scavenging activities and ferric reducing power are most commonly used. However, the total antioxidant activity of an antioxidant cannot be evaluated by using one single method, due to oxidative processes. Therefore, at least two methods should be employed in order to evaluate the total antioxidant activity (Gulcin *et al.*^2005^). Present study was undertaken to demonstrate the antioxidant capacity of stem bark extract of *S. roxburghii* by vitro methods. *Shorea* species are well known for its phenolic content and antioxidant activities. Norizan *et al.* (2012) have reported the phenolic content of *Shorea acuminate, Shorea leprosula, Shorea resinosa, Shorea macroptera* and *Shorea bracteolate* (2731, 2615, 2461, 2461 and 2423 mg/100 g). Similarly they reported the total antioxidant property of the methanol extracts in the following order: *Shorea macroptera> Shorea leprosula> Shorea resinosa> Shorea acuminate>Shorea bracteolate* at 98.68, 78.42, 71.11, 57.47 and 56.75%, repscetively. Recently, Wani *et al.* (2012) reported the wound healing capacity of ethanolic extract of *Shorea robusta. Shorea* species are rich in stilbenes which are made up of resveratrol derivatives, are highly bioactive compounds. Many authors have reported several bioactive phenolic compounds from *S. roxburghii.* Roxburghiol A, Melanoxylin A, Caragaphenol A, (−)-ε-viniferin, Hopeahainanphenol, Vitisinol G, Vaticanol A, (−)-hopeaphenol, Isohopeaphenol, Apigenin 7-O-arabinoside, trans-piceid, and trans-3, 5, 4^′^-trihydroxy resveratrol 2-C-glucoside from the bark of *S. roxburghii* has been reported by Patcharamun *et al.*^2011^isolated*.* Similarly, Morikawa *et al.* (Morikawa *et al.*^2012^& Morikawa *et al.*^2012^) have reported resveratrol, piceid, trans-resveratrol 10 C-β-D-glucopyranoside, Cis-resveratrol 10-C-ß-D-gluco pyranoside, Phayomphenols A_1_, Phayomphenols A_2_, S-Dihydrophayomphenol A_2_, Phayomphenol B_1_, Phayomphenol B_2_ (3), (−)-Ampelopsin A, Hopeafuran, (−)-Balanocarpol, Malibatols A, Malibatol B, Vaticanol A, Vaticanol E, Vaticanol G, (+)-Parviflorol. (−)-α-Viniferin, (−)-Ampelopsin H and Hemsleyanol D. Resveratrol and its derivatives are powerful antioxidants (Pour Nikfardjam *et al.*^2006^). This much higher activity may be due to the presence of above mentioned high molecular weight phenolic compounds which are resveratrol derivatives, have number of aromatic rings and hydroxyl groups. With respect to biological activities, only scared studies are undertaken in *Shorea roxburghii.* It can be seen that, the solvents acetone and methanol are suitable for extraction of antioxidant compounds present in *S. roxburghii* since the radical scavenging activity of acetone and methanol extracts are similar.

### Synthesis of silver nanoparticles

Synthesis of silver nanoparticles has drawn much attention due to its vast application in various fields. Silver nanoparticles are used in the field of magnetics, optoelectronics, information storage (Okuda *et al.*^2005^; Dai and Bruening, 2002; Murray *et al.*^2001^), catalysis (Watanabe *et al.*^2006^), biosensing, imaging, drug delivery, nanodevice fabrication and medicine (Nair and Laurencin, 2007; Lee and El-Sayed, 2006; Jain *et al.*^2008^). Various methods such as chemical (Sun *et al.*^2002^), electrochemical (Yin *et al.*^2003^), radiation (Dimitrijevic *et al.*^2001^, photochemical (Callegari *et al.*^2003^) and biological methods (Naik *et al.*^2002^) are preferred for synthesis of silver nanoparticles. The biosynthetic method employing plant extracts has drawn attention as a simple and viable alternative to chemical procedures and physical methods. Even though numbers of physical and chemical methods are available for the synthesis of silver nanoparticles, they could create problems owing to the use of toxic solvents, generation of by-products and high energy consumption. Hence, there is a constant search to develop environmentally benign procedures for the synthesis of silver nanoparticles. Recently, *Murraya koenigii* leaf (Philip *et al.*^2011^), *Mangosteen* leaf (Veerasamy *et al.*^2011^), *Mangifera indica* leaf (Philip, 2011), Tansy fruit (Dubey *et al.*^2010^), *Jatropha curcas* (Bar *et al.*^2009^), *Cinnamomum zeylanicum* leaf (Smitha *et al.*^2009^), *Camellia sinensis* (Nestor *et al.*^2008^), Aloe vera (Chandran *et al.*^2006^), Mushroom extracts (Philip, 2009) and Honey (Philip, 2009) have been used for the synthesis of metal nanoparticles.

The kinetics of the reaction between silver ions and stem bark extracts of *S. roxburghii* was monitored by recording the absorption spectra as a function of time. By employing the variable volume of extract (1.0, 2.5, 3.5 and 5.0 mL) with 1.0 mM silver nitrate, the effect of concentrations of the extract on the rate of bioreduction was studied. Adding separately different volume of extract of *S. roxburghii* to the silver nitrate solution, a characteristic sharp surface plasmon resonance (SPR) band was appeared from 412–432 nm indicating the formation of Ag nanoparticles.

**Figure 2:**
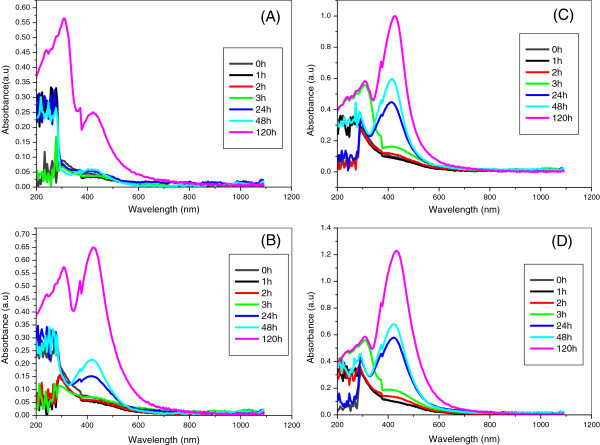
**UV-Visible spectra of AgNPs synthesized from 1.0 mM silver nitrate at various time interval (A) 1.0 mL; (B) 2.5 mL; (C) 3.5 mL; (C) 5.0 mL extract of*S. roxburghii.***

Figure 2 (A-D) show the UV-Visible absorption spectrum of the AgNPs as a function of the concentrations, 1.0, 2.5, 3.5 and 5 mL of extract with 1 mM AgNO_3._ Neither yellowish-brown color change in the reaction vessel nor a strong plasmon resonance peak was observed for the silver nitrate solution, which was mixed with the water extract of *S. roxburghii* at 0 h. Upon addition of the extract to the silver nitrate solution, the color of the solution was changed from yellow to brown due to the reduction of Ag^+^ to metallic Ag^0^. It is well known that silver nanoparticles exhibit yellowish brown color in water, which arises due to the excitation of surface plasmon resonance in the metal nanoparticles (Nand and Saravanan, 2009). The relationship between the surface plasmon resonance, metal nanoparticle’s size and shape is well established (Wiley *et al.*^2006^). Metal nanoparticles such as silver have free electrons, which give rise to surface plasmon resonance absorption band (Noginov *et al.*^2006^). Consequently size and shape of nanoparticles in aqueous suspension can be judged by UV-visible absorbance studies. This important observation indicates that the reduction of the Ag^+^ ions takes place in the extract of *S .roxburghii* under visible-light irradiation. The increase in intensity is due to increasing the concentration of silver nanoparticles formed as a result of reduction of silver ions present in the aqueous solution.

**Figure 3:**
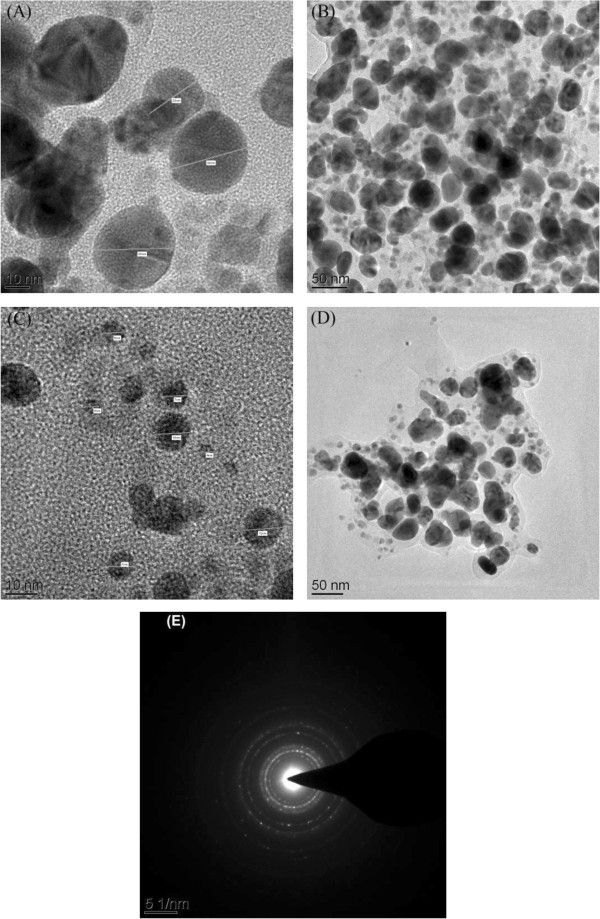
**TEM images of synthesized AgNPs. (A)**; **(B)**; **(C)**; **(D)** TEM image of AgNPs; **(F)** SEAD of AgNPs.

Upon addition of 1.0 mL of extract with 1 mM silver nitrate solution, λ_max_ or intensity was observed at 24 h followed by 48 and 120 h. The lower volume of extracts (1.0 & 2.5 mL) was insufficient to produce AgNPs as it requires 24 h to reduce the silver ions. Nucleation of silver nanopaticles was started only after 2 h during the addition of 3.5 and 5.0 mL of extract which was found to adequate to reduce the silver ions. The concentrations, 3.5 and 5.0 mL of extract was sufficient to produce the silver nanoparticles, but there was a sharp difference between these two particularly with respect to intensity. SPR peak resulted from 5.0 mL extract has high intensity (1.229 a.u) whereas 3.5 mL has low intensity (1.000 a.u). The high intensity of the peak is due to the high concentration of silver nanoparticles. From the UV-Visible studies, it has been found that the amount of the extract has played a vital role in the formation of AgNPs. It has been proved that 5.0 mg of the extract is enough to produce silver nanoparticles. The TEM analysis was also carried out for AgNPs synthesized using 5.0 mL of extract to find out the shape and size of the particles. Figure 3 (A), (B), (C) and (D) depict the typical TEM images of synthesized AgNPs. These pictures exhibit that the majority of the particles are in spherical shape with smooth surfaces. Figure 3 (F) shows the selected area electron diffraction (SAED) and suggests the polycrystalline nature of the green synthesized AgNPs. The TEM image represents the frequency of TEM size distribution of AgNPs ranging from 4–50 nm.

The reducing capacity depends on the amount of water soluble phenolic compound present in the extract. During the reaction with silver nitrate, the phenolic compound donates electron to Ag^+^ to produce Ag^0^. After donation of an electron, the phenolic compounds changed into quinine which is stabilized by the resonance structure of the same. The bioreduction of silver ions and the formation of AgNPs are closely related to the biomolecular component of the extract. Biosynthesis is a green process, no by-products and wastage formed during the reaction. Several authors reported green synthesis using various medicinal plants with different shape and sizes.

## Conclusions

We have demonstrated that the stem bark extracts of *Shorea roxburghii* contain high level of total phenolic compounds and radical scavenging activity. Acetone extract showed highest scavenging activities against DPPH and hydroxyl radicals. Methanol extract showed highest activities against ABTS, hydrogen peroxide radicals and ferric reducing power. The stem bark extract of *S. roxburghii* can be explored for its applications in the prevention of free radical related diseases.

Shorea roxburghii stem bark extract have been effectively used for the synthesis of silver nanoparticles. We have demonstrated the use of a natural, renewable and low-cost bioreducing agent. This plant extract could be used as an efficient green reducing agent for the production of AgNPs. The spectroscopic characterization from UV-Visible and TEM supports the stability of the biosynthesized nanoparticles. The size of the particles is found to be 4–50 nm.
